# Testosterone Therapy and Tendon Injury: Clinical Evidence, Biological Mechanisms, and Sports Medicine Implications

**DOI:** 10.7759/cureus.114876

**Published:** 2026-08-20

**Authors:** Khashayar Farzam

**Affiliations:** 1 Metabolic Health, True North Metabolic, Kitchener, CAN; 2 Health Sciences, McMaster University, Kitchener, CAN

**Keywords:** androgen exposure, hypogonadism, musculoskeletal safety, resistance training, rupture

## Abstract

Testosterone therapy increases lean mass and can improve muscle strength in men with hypogonadism. This review examines the relationship between testosterone therapy, tendon adaptation, and tendon injury, including the possibility of a mismatch between changes in muscle strength and tendon capacity. This narrative review critically evaluates the clinical and experimental evidence relating testosterone exposure to tendon injury. The present review focuses specifically on tendon-related safety and is not intended to assess the overall benefit-risk profile of testosterone therapy.

Prescription-based studies have identified associations with distal biceps, rotator cuff, quadriceps, patellar, and Achilles tendon injuries. The magnitude of association varies considerably, with modest elevations in broad analyses and larger relative estimates in some site-specific studies. Absolute event rates are generally low, and the available studies cannot establish causation. Important limitations include incomplete information about treatment indications, testosterone concentrations, dosage, adherence, formulation, physical activity, pre-existing tendinopathy, and undisclosed anabolic-androgenic steroid use.

Evidence from supraphysiologic androgen exposure provides greater biological plausibility but cannot be directly extrapolated to physiologic testosterone replacement. Potential biological mechanisms involving collagen synthesis, matrix remodelling, and tendon mechanical properties are also examined.

No clinical evidence establishes a dose-response relationship or demonstrates that topical treatment, smaller injections, or more frequent dosing reduces tendon risk. Testosterone improves bone mineral density and estimated bone strength, but fracture prevention has not been demonstrated. Overall, a small increase in tendon injury risk with testosterone therapy is plausible, particularly in highly active individuals undergoing rapid increases in loading, but the evidence remains observational and substantially confounded. Clinicians should provide proportionate counselling without discouraging appropriately indicated treatment.

## Introduction and background

Male hypogonadism is a clinical syndrome caused by inadequate testicular testosterone production, impaired hypothalamic or pituitary stimulation, or both. The diagnosis requires compatible symptoms or signs together with consistently low morning testosterone concentrations, rather than an isolated biochemical measurement [1,2]. Testosterone therapy is used to restore androgen concentrations and improve clinically relevant manifestations such as sexual symptoms, reduced lean mass, anemia, and impaired bone density. The distinction between organic hypogonadism and lower testosterone associated with obesity, chronic illness, medication exposure, aging, or other potentially reversible factors remains important, although that broader controversy is outside the scope of this review.

Testosterone prescribing has changed considerably over the past two decades. United States prescribing increased substantially during the early 2000s, declined following regulatory and cardiovascular safety concerns, and subsequently increased again in several prescribing datasets [3,4]. This expansion has increased the number of treated men who participate in resistance training, recreational sport, and physically demanding occupations. It has also created a larger population in which uncommon musculoskeletal events may be detected.

Testosterone has well-established anabolic effects on skeletal muscle. Physiologic replacement increases lean mass and muscle size in hypogonadal men, while progressively higher exposures produce dose-dependent increases in muscle volume, strength, and power [5,6]. Tendon tissue also adapts to mechanical loading, but its response is governed by collagen turnover, extracellular matrix organization, cross-sectional area, and changes in material properties [7-9]. These adaptations may not occur at the same rate as improvements in muscle performance. This has led to the hypothesis that testosterone could increase tendon injury risk either through direct effects on tendon tissue or indirectly by permitting force production and training loads to increase faster than tendon capacity.

Interest in this possibility has grown after several insurance-claims studies reported associations between prescription testosterone and injuries involving the distal biceps, rotator cuff, quadriceps, patellar tendon, and Achilles tendon [10-16]. These reports require careful interpretation. Prescription testosterone is not equivalent to supraphysiologic anabolic-androgenic steroid exposure, and an association recorded in an administrative database does not establish a pharmacologic cause. Accordingly, this review examines the magnitude and limitations of the reported association between testosterone therapy and tendon injury, evaluates the biological plausibility of this association, and considers its implications for sports medicine practice; it is not intended as a comprehensive assessment of the overall benefits and risks of testosterone therapy.

## Review

Review approach

A focused narrative search of PubMed and MEDLINE was conducted through August 2026. Search terms included combinations of testosterone, testosterone therapy, hypogonadism, anabolic-androgenic steroids, tendon, rupture, tendinopathy, collagen, extracellular matrix, rotator cuff, biceps, quadriceps, patellar tendon, Achilles tendon, pharmacokinetics, bone density, and fracture. Human observational studies, controlled trials, experimental studies, pharmacokinetic investigations, and clinically relevant reviews were considered. Reference lists of key publications were also examined.

Studies were selected if they directly evaluated testosterone or anabolic-androgenic steroid exposure in relation to tendon injury, tendon structure or biomechanics, tendon healing, or clinically relevant musculoskeletal outcomes, or if they provided mechanistic evidence relevant to these associations. Studies were prioritized when they included human clinical outcomes, defined testosterone exposure, or directly evaluated tendon tissue. Animal and experimental studies were included when they addressed potential biological mechanisms. References of relevant articles were also reviewed for additional studies. Study identification, selection, and data extraction were performed by the sole author. Because this was designed as a narrative rather than systematic review, no formal risk-of-bias instrument or Preferred Reporting Items for Systematic Reviews and Meta-Analyses (PRISMA) methodology was applied.

The evidence was not sufficiently homogeneous for meta-analysis. Clinical studies differed in their definitions of testosterone exposure, tendon outcomes, follow-up intervals, matching procedures, and populations. Particular emphasis was therefore placed on absolute event rates, outcome definitions, consistency across anatomical sites, exposure measurement, and the ability of each design to address causation.

Tendon adaptation and the basis for concern

Tendons are hierarchical collagen-rich structures that transmit force from muscle to bone. Type I collagen predominates, while smaller quantities of type III collagen, proteoglycans, elastin, and other matrix components influence fibril organization, interfascicular sliding, and viscoelastic behavior. Mechanical loading stimulates tendon collagen synthesis, but increased synthesis does not immediately produce a mature, load-bearing matrix. Collagen must be assembled, aligned, cross-linked, and incorporated into the existing structure.

Human exercise studies demonstrate coordinated increases in muscle and tendon protein synthesis after loading [7]. The time course of functional adaptation, however, is not identical. In a resistance-training study, strength and neural activation improved before statistically significant changes in muscle cross-sectional area and tendon stiffness were observed [8]. Achilles tendon collagen markers, imaging characteristics, and stiffness also required approximately two to three months of training to change measurably in another small study [9]. These findings do not prove that testosterone creates a muscle-tendon mismatch, but they make the concept biologically credible when muscle strength or training load rises quickly.

This issue is most relevant in individuals who begin testosterone and simultaneously increase resistance-training volume, return to sport, gain body mass, or resume high-force activity after a period of deconditioning. In such cases, any risk may arise from the combined change in loading environment rather than a direct toxic effect on collagen. Claims databases generally cannot distinguish this scenario from ordinary replacement therapy without a change in activity.

Clinical evidence involving prescription testosterone

Broad Analyses of Tendon Tears

The broadest recent analysis evaluated 112,242 men who initiated testosterone after a primary care visit without documentation of laboratory criteria supporting treatment. They were compared with 448,968 matched controls [10]. Testosterone exposure was associated with higher odds of any tendon tear at one and two years, with odds ratios of 1.33 and 1.31, respectively. Rotator cuff and patellar tendon tears were also more frequent, as were several tendon repair procedures. The consistency across one-year and two-year analyses supports a signal, but the magnitude was modest.

The study’s inclusion criterion is also relevant. It selected men without documented laboratory evidence meeting the investigators’ definition of treatment eligibility. This does not establish that each prescription was clinically inappropriate, because laboratory tests performed outside the captured system may have been missed. It may nevertheless identify a population with more heterogeneous indications, less standardized follow-up, or greater use for body composition and performance-related goals. Those factors could affect physical activity and injury risk independently of testosterone, which is a significant confounding variable.

Distal Biceps Injury

A matched analysis of 291,610 testosterone-exposed patients and an equal number of controls found 650 distal biceps injuries in the testosterone group and 159 in the control group during the first year [11]. This produced an odds ratio of 4.10. Over the full follow-up period, the association was smaller, with an odds ratio of 2.07. Among men, testosterone exposure was also associated with a greater likelihood of surgical repair following injury.

The relative estimate is striking, but the absolute one-year risks were approximately 0.22% and 0.05%. The absolute difference was therefore roughly 0.17 percentage points. Distal biceps rupture is strongly influenced by age, resistance training, occupational loading, smoking, body mass, and pre-existing degeneration. Matching addressed some comorbidities but could not account for training behavior, maximal strength, arm circumference, previous tendon symptoms, or nonprescribed androgen use.

The inclusion of women in the overall cohort further complicates clinical interpretation because indications, doses, and exposure patterns may differ from those of men treated for hypogonadism. The male-specific analysis produced a smaller effect estimate than the overall one-year analysis, which argues against applying the largest odds ratio directly to routine male replacement therapy.

Quadriceps and Patellar Tendon Injury

A separate claims analysis matched 151,797 testosterone-exposed patients with an equal number of controls [12]. Within one year, 97 exposed patients and 18 controls received a diagnosis classified as a quadriceps injury, corresponding to rates of 0.06% and less than 0.01%. The odds ratio was 5.4 overall and 5.8 among men.

Although the relative difference was large, the outcome was rare and included coding for quadriceps muscle or tendon injury rather than only imaging-confirmed tendon rupture. A small number of miscoded or clinically dissimilar events could therefore materially influence the estimate. The study also reported increased odds of tendon repair among those with injury, but analyses conditioned on receiving an injury diagnosis can introduce additional selection. More active patients may be both more likely to rupture a tendon and more likely to pursue surgery.

The broader study by Waters et al. found smaller associations for quadriceps and patellar tendon outcomes [10]. Quadriceps tendon tears were elevated at one year, with an odds ratio of 1.59, while patellar tendon tears had odds ratios slightly above 2 at one and two years. These estimates may be more representative of the overall treated population, although the same limitations surrounding exposure and activity remain.

Rotator Cuff Outcomes

A large database study reported a rotator cuff tear rate of 1.14% among testosterone-treated patients compared with 0.19% among controls, with an adjusted odds ratio of 3.57 [13]. Testosterone-treated patients with a rotator cuff tear were also more likely to undergo repair. The investigators reported a particularly large association with a subsequent rotator cuff operation within one year of the first repair.

Several aspects of these findings warrant caution when interpreting the data. Rotator cuff disease often develops gradually and may be asymptomatic before diagnosis. Increased health-care contact, shoulder imaging, or willingness to undergo surgery could increase recorded events without a comparable increase in newly developed structural tears. The subsequent repair outcome was recorded irrespective of laterality, so it may have included contralateral procedures and staged treatment rather than true revision of a failed repair.

More recent postoperative analyses have not been entirely consistent. One matched study found no significant difference in two-year revision rotator cuff repair rates between preoperative testosterone users and controls, although small differences in other shoulder outcomes were reported [14]. These conflicting postoperative findings underscore the limitations of procedure codes as a substitute for tendon healing, structural integrity, or recurrent rupture.

An additional complication is that low testosterone itself has been associated with rotator cuff repair. In a large insurance analysis, men coded as testosterone deficient had higher odds of undergoing rotator cuff repair than men without that diagnosis [15]. This does not prove that testosterone deficiency causes rotator cuff disease, but it illustrates the potential for confounding by indication. Men selected for testosterone treatment may already have a different musculoskeletal risk profile before therapy begins.

Achilles Tendon Injury

A matched study of 423,278 testosterone-treated patients and an equal number of controls evaluated Achilles tendon injury over two years [16]. The incidence was 377.8 per 100,000 person-years in the testosterone group and 245.8 per 100,000 person-years in controls. After adjustment, testosterone exposure was associated with an odds ratio of 1.24. Among patients diagnosed with an Achilles injury, surgery occurred in 9.0% of the testosterone group and 6.4% of controls.

This is among the more clinically interpretable findings because the relative association was modest and the authors reported an estimated number needed to harm of 371 over the study interval. Even this estimate should not be regarded as a causal number needed to harm. The calculation assumes that the adjusted difference results from testosterone itself, while unmeasured activity, body mass, prior tendinopathy, medication use, and diagnostic surveillance could account for part of the difference.

Evidence against a large generalized injury effect

Not all population evidence among those undergoing testosterone therapy supports a broad increase in tendon injury. A self-matched Ontario study evaluated 64,386 adults before and after testosterone initiation [17]. Rates of serious injuries requiring emergency care were nearly identical before and after treatment, with a relative risk of 1.00. The result was consistent across formulations and injury mechanisms.

This study was not designed specifically for tendon rupture and could miss smaller site-specific associations. Its self-matched design nevertheless reduces confounding from stable patient characteristics. The absence of an increase in serious injury suggests that testosterone does not produce a large generalized rise in traumatic events. Any tendon-specific effect is more likely to be limited in absolute magnitude or concentrated in particular exposure and activity patterns. The principal clinical studies evaluating prescription testosterone and tendon-related outcomes are summarized in Table 1.

**Table 1:** Principal clinical studies evaluating prescription testosterone and tendon-related outcomes OR, odds ratio; RR, relative risk; CI, confidence interval.

Study	Population and testosterone exposure	Outcome and follow-up	Absolute event rate	Effect estimate	Principal limitations
Waters et al. [10]	112,242 men initiating testosterone within 30 days of a primary care visit, matched to 448,968 controls; cohort excluded men with documented diagnoses meeting the study definition for treatment of low testosterone	Any tendon tear, rotator cuff tear, quadriceps tendon tear, and patellar tendon tear at 1 and 2 years	At 1 year, rotator cuff tear occurred in 0.743% of testosterone-treated patients versus 0.539% of controls	Any tendon tear at 1 year: OR 1.33 (95% CI 1.23-1.44); rotator cuff tear: OR 1.35 (95% CI 1.25-1.46); quadriceps tendon tear: OR 1.59 (95% CI 1.05-2.40); patellar tendon tear: OR 2.06 (95% CI 1.10-3.86)	Administrative claims data; no serum testosterone concentrations, dose, adherence, or physical activity data; treatment indication may have been incompletely captured; differences in glucocorticoid and fluoroquinolone exposure remained between cohorts
Rebello et al. [11]	291,610 testosterone-exposed patients matched 1:1 to controls; testosterone prescription filled for at least 3 consecutive months	Distal biceps tendon injury within 1 year and over total follow-up	650/291,610 (0.22%) versus 159/291,610 (0.05%) within 1 year	OR 4.10 (95% CI 3.45-4.89) at 1 year; OR 2.07 (95% CI 1.94-2.38) over total follow-up	Claims-based outcome ascertainment; cohort included men and women; training exposure, strength, pre-existing tendon disease, testosterone concentrations, and nonprescribed androgen use unavailable
Meghani et al. [12]	151,797 testosterone-exposed patients matched 1:1 to controls	Quadriceps muscle or tendon injury within 1 year	97/151,797 (0.06%) versus 18/151,797 (<0.01%)	OR 5.4 (95% CI 3.4-9.2); male subgroup OR 5.8 (95% CI 3.5-10.3)	Very low absolute event rate; outcome codes included quadriceps muscle and tendon injuries; no dose, serum concentration, physical activity, or nonprescribed androgen data
Testa et al. [13]	Patients prescribed testosterone for at least 90 days in the PearlDiver database	Rotator cuff tear, repair, and subsequent repair	Rotator cuff tear: 1.14% in testosterone-treated patients versus 0.19% in controls	Adjusted OR for rotator cuff tear 3.57 (95% CI 3.57-3.96); adjusted OR for repair 1.60 (95% CI 1.54-1.67)	Claims-based diagnosis; rotator cuff disease may predate diagnosis; surveillance and treatment-selection bias; subsequent repair was recorded irrespective of laterality
Lawand et al. [14]	5,109 preoperative testosterone users matched 1:1 to 5,109 controls undergoing arthroscopic rotator cuff repair	Postoperative complications and revision rotator cuff repair through 2 years	Revision rotator cuff repair: 2.6% versus 2.3%	No significant difference in revision repair, P = 0.41	Postoperative rather than incident tendon-injury study; claims data; exposure defined by preoperative prescription; cannot directly assess tendon healing or structural re-tear
Albright et al. [16]	423,278 patients prescribed testosterone for at least 3 consecutive months matched 1:1 to controls	Achilles tendon injury and subsequent surgery over 2 years	Achilles injury: 0.76% versus 0.49%; surgery among injured patients: 9.0% versus 6.4%	Adjusted OR for Achilles injury 1.24 (95% CI 1.15-1.33); surgery after injury OR 1.54 (95% CI 1.19-1.99)	Administrative claims data; dose, route, adherence, serum testosterone concentrations, and activity level unavailable; residual confounding despite matching
Yarnell et al. [17]	64,386 adults initiating testosterone in Ontario; self-matched comparison of periods before and after treatment	Serious traumatic injury requiring emergency medical care	34,439 injuries before treatment and 7,349 after treatment; 584 versus 565 injuries per month	RR 1.00 (95% CI 0.96-1.04)	Outcome was serious injury broadly rather than tendon injury specifically; self-matched design reduces stable confounding but cannot exclude time-varying confounding

Evidence from supraphysiologic androgen exposure

The association between anabolic-androgenic steroid use and tendon rupture is more established than the evidence for physiologic replacement, although it remains based largely on observational data. In a cohort of 142 experienced male recreational and competitive bodybuilders, 22% of long-term anabolic-androgenic steroid users reported at least one lifetime tendon rupture compared with 6% of nonusers [18]. The hazard ratio for a first rupture was 9.0. Upper-body ruptures occurred exclusively among users, whereas lower-body rupture rates did not differ significantly.

This upper-body distribution is clinically notable. Pectoralis major, distal biceps, and triceps ruptures are repeatedly described in strength athletes using supraphysiologic androgens. These tendons experience high forces during pressing and pulling movements, and marked androgen-associated gains in upper-body strength may contribute. Case series of pectoralis major rupture in bodybuilders provide additional clinical context but cannot separate drug exposure from extreme training loads, exercise technique, accumulated degeneration, and selection bias [19]. Anecdotal reports also exist in powerlifting.

Human imaging and tissue studies provide a less uniform picture. Resistance-trained anabolic-androgenic steroid users had greater patellar tendon stiffness and Young’s modulus than trained nonusers in one study, despite both trained groups having larger and stiffer tendons than untrained controls [20]. Greater stiffness is not inherently pathological, but a tendon that deforms less under load may have less capacity to absorb energy before failure.

A 2025 study of current users, former users, and nonusers found a substantially greater reported prevalence of upper-body tendon injury among the combined androgen-exposed group [21]. However, patellar tendon cross-sectional area, cell density, and most measured tissue characteristics were not markedly different. Former users demonstrated greater deformation and strain at a common force. Greater deformation and strain at a given force reflect greater tendon compliance and should not, in isolation, be interpreted as evidence of tendon damage. These findings suggest that clinically relevant effects may not be captured by tendon size or a single biopsy site, and that upper-body injury history does not necessarily correspond to severe persistent abnormalities in the patellar tendon.

Human histology data is sparse. An ultrastructural examination of ruptured tendon from two anabolic steroid users and two nonusers found no clear difference in collagen fibril appearance [22]. A single case report described distal biceps rupture during long-term androgen substitution, but it cannot establish causality [23]. Overall, the human supraphysiologic literature supports an association with rupture, particularly in the upper body, but provides limited evidence about a specific microscopic lesion.

The anabolic effects of high-dose testosterone are unquestionably greater than those of replacement therapy. In a controlled trial, 600 mg of testosterone enanthate weekly increased muscle size and strength, with the largest changes occurring when testosterone was combined with resistance training [24]. These exposures and responses should not be treated as equivalent to properly titrated replacement. They do, however, support a continuum in which muscle force, training tolerance, and tissue exposure become more relevant as dose and achieved concentration rise.

Is there evidence of a dose-response relationship?

A direct human dose-response relationship between testosterone exposure and tendon injury has not been demonstrated. The available prescription studies generally identify a filled prescription, a minimum treatment duration, or a coded treatment history. They do not report the dispensed weekly dose in a clinically interpretable manner, achieved testosterone concentrations, timing of blood sampling, adherence, or whether additional nonprescribed androgens were used. The latter point of nonprescribed androgens, such as trenbolone or stanozolol, is key as the usage of these substances is very common among those using higher doses of testosterone for recreational use or performance reasons.

Indirect evidence suggests that dose could matter. Skeletal muscle mass and strength increase in relation to testosterone dose and circulating concentration [6]. If tendon adaptation does not accelerate proportionately, a greater mismatch could occur at higher exposures. The controlled bodybuilding literature also involves doses far above replacement and shows a stronger rupture signal than the prescription literature [18,24,25].

Animal studies provide the clearest, although still inconsistent, suggestion of exposure dependence. In rats, reductions in Achilles tendon collagen-biosynthesis markers and hydroxyproline were observed after three weeks only with a dose five times the therapeutic level, not with the therapeutic dose [26]. Other experiments found that anabolic steroid exposure produced tendons that were stiffer and failed with less elongation, while peak strength was unchanged [27,28]. The changes reported by Inhofe et al. were reversible after discontinuation, suggesting altered material behavior rather than permanent destruction [28].

Mechanical loading may modify these effects. Nandrolone exposure combined with exercise reduced matrix metalloproteinase activity and altered Achilles tendon remodelling in rats [29]. Different tendons showed reduced compliance after nandrolone exposure, with some effects amplified by concurrent jump training [30]. Another experiment found that nandrolone reversed favorable exercise-associated changes in Achilles tendon failure stress and produced areas of collagen dysplasia [31]. Earlier ultrastructural work also described disorganized or dysplastic collagen fibrils following anabolic steroid administration [32].

These studies differ in species, compound, dose, duration, loading model, and biomechanical testing. Some show reduced compliance or energy absorption, while others show minimal changes in ultimate strength or morphology. A review of the experimental literature similarly concluded that results are heterogeneous and frequently contradictory [25]. Animal findings therefore support biological plausibility but cannot define a safe threshold or predict the effect of routine testosterone replacement in humans.

Potential biological mechanisms

Muscle-Tendon Adaptation Mismatch

One proposed mechanism is an indirect mismatch between increasing muscle force and slower tendon adaptation. Testosterone can improve lean mass, muscle protein synthesis, and strength over months [5,6]. A patient who feels stronger or recovers more effectively may increase weight, repetitions, frequency, or training intensity. Tendon remodeling may lag behind these changes, particularly after previous inactivity or chronic hypogonadism.

This mechanism does not require direct tendon toxicity. It predicts that the risk would be greatest when testosterone initiation coincides with rapid training progression. It may also help explain why upper-body ruptures are prominent among anabolic steroid users, since pressing and pulling strength may increase rapidly and expose the pectoralis major, distal biceps, and triceps tendons to high eccentric forces.

Collagen Synthesis and Organization

High-dose animal studies suggest that androgens can transiently suppress selected collagen-synthesis markers [26]. Other models describe altered crimp morphology, fibril organization, or collagen dysplasia [31,32]. These structural findings are not universal, and the small human ultrastructural study did not identify comparable changes [22].

Exercise normally increases collagen synthesis in tendons [7,33]. An androgen-mediated disturbance in the timing, organization, or maturation of newly synthesized collagen could theoretically impair adaptation even if total collagen production is not chronically reduced [34]. This remains speculative because human tendon collagen turnover has not been measured prospectively during testosterone replacement.

Matrix Metalloproteinases and Remodelling

Matrix metalloproteinases participate in extracellular matrix turnover and allow damaged or mechanically unsuitable collagen to be removed and replaced. In rat Achilles tendon, nandrolone combined with loading inhibited matrix metalloproteinase activity and altered remodelling [29]. A reduction in matrix turnover could produce a tendon that is mechanically stiff yet less capable of accommodating repeated loading or repairing microscopic damage.

The direction of effect may depend on timing. Excessive matrix degradation can also weaken tendons, while controlled inhibition might theoretically aid healing in some circumstances. This complexity makes a single “reduced collagen” explanation inadequate.

Stiffness, Elongation, and Energy Absorption

Several experimental studies report increased stiffness, reduced elongation before failure, or reduced energy absorption after anabolic steroid exposure [27,28,30]. A stiffer tendon may transmit force efficiently but tolerate less deformation before rupture. Material stiffness must also be distinguished from structural stiffness, which is influenced by tendon dimensions. A larger tendon can be structurally stiff without having intrinsically abnormal tissue properties.

Human data are compatible with, but do not establish, this mechanism. Patellar tendon modulus and stiffness were higher in anabolic steroid users than in trained nonusers [20]. More recent work found relatively limited abnormalities in current users and greater deformation in former users [21]. These differences may reflect exposure timing, specific drugs, cumulative dose, tendon selection, or training history.

Effects on healing may differ from effects on uninjured tendon

Androgen effects are not uniformly harmful. In a murine rotator cuff repair model, testosterone supplementation improved aspects of tendon healing and functional recovery [35]. Experimental anabolic agents have also been investigated as potential treatments for muscle loss following chronic tendon injury. An androgen could therefore have different effects on an acutely healing tendon, a degenerative tendon, and a healthy tendon undergoing repetitive loading.

This distinction may help explain why the postoperative human literature is inconsistent. Database analyses of revision operations are influenced by surgical selection, tear size, rehabilitation, laterality, and health-care utilization. They should not be interpreted as direct measures of tendon-to-bone healing.

Formulation, injection frequency, and hormonal stability

No clinical study has established that tendon injury risk differs between injectable, transdermal, oral, intranasal, or implanted testosterone. The large claims studies generally combine formulations or do not provide route-specific tendon outcomes. There is also no direct evidence comparing weekly injections with smaller, more frequent injections.

Formulations have different pharmacokinetic profiles. In a randomized comparison, a transdermal patch maintained testosterone within physiologic ranges with a circadian pattern, whereas 200 mg of intramuscular testosterone enanthate every two weeks produced supraphysiologic testosterone levels for several days after each injection [36]. Transdermal patch and gel preparations also produce different profiles. A patch may approximate a circadian pattern, while gel produces a flatter average profile but can generate variable individual peaks [37].

It is reasonable to ask whether avoiding high peaks could reduce tendon effects. A peak concentration might transiently increase muscle performance, training drive, fluid retention, or other androgen-responsive processes. Smaller, more frequent injections may reduce peak-to-trough variation compared with large, infrequent doses. Topical therapy may also avoid the pronounced early peak associated with some intramuscular regimens.

These ideas remain theoretical. No study has shown that hormonal stability improves tendon collagen turnover, reduces tendon stiffness, or prevents injury. Conversely, no study has shown that ordinary injection peaks cause tendon rupture. Injection frequency is also correlated with dose, patient preference, monitoring intensity, athletic behavior, and prescriber practice. A route-specific recommendation for tendon safety would therefore be premature.

The practical objective remains restoration of physiologic testosterone concentrations while avoiding sustained supraphysiologic exposure. Formulation should be selected according to clinical response, pharmacokinetics, patient preference, cost, adverse effects, and the ability to monitor treatment, not an unproven difference in tendon risk.

Confounding and the problem of causal inference

The current clinical literature is vulnerable to several overlapping forms of confounding. Physical activity is the most important factor. Individuals receiving testosterone may be more likely to participate in resistance training, bodybuilding, recreational sport, or physically demanding employment. Even within the same activity, they may lift heavier loads or progress more quickly than controls. Claims databases do not measure these exposures. Ultimately, there has to be an acute provoking factor for tendon injury to occur and physical activity will often take that role.

Body composition also matters. Obesity and higher body mass increase loading across the lower extremity and have been associated with Achilles tendon problems [38]. Diabetes is associated with collagen glycation, altered tendon structure, and rotator cuff abnormalities [39]. Smoking, dyslipidemia, chronic kidney disease, inflammatory disease, and aging may influence tendon degeneration. Although propensity matching captures coded diagnoses, it does not reliably measure disease severity, duration, metabolic control, or uncoded risk factors.

Medication exposure is another limitation. Fluoroquinolones are associated with tendon injury, particularly involving the Achilles tendon [40]. Systemic or locally administered corticosteroids may also affect tendon integrity. Database studies differ in how completely they account for these drugs and may not capture prescriptions outside the network.

Confounding by indication is especially difficult. Men with organic hypogonadism may have reduced muscle mass, poorer bone health, chronic illness, or prolonged inactivity before treatment. Men receiving testosterone for functional symptoms may have obesity, sleep disturbance, or metabolic disease. Others may seek testosterone partly because they are highly invested in exercise performance. These groups are not interchangeable, yet prescription coding may combine them.

Exposure misclassification further weakens causal inference. A filled prescription does not prove that medication was taken, and a control designation does not exclude testosterone obtained from another source. Administrative records rarely provide the actual weekly dose, serum concentrations, or duration of supraphysiologic exposure. Undisclosed anabolic-androgenic steroid use could be particularly important in highly muscular or strength-trained individuals.

Outcome coding is similarly imperfect. Tendinitis, partial tearing, complete rupture, muscle strain, and postoperative diagnosis codes may be grouped together. Some studies assess surgical repair rather than injury itself. Surgery depends on age, activity expectations, tear chronicity, insurance, access, surgeon preference, and willingness to undergo rehabilitation.

The studies also do not represent wholly independent replication. Several used the same or similar commercial databases, overlapping years, related coding strategies, and overlapping investigative groups. Similar results across anatomical sites are informative, but shared methods can reproduce shared biases. Commentary on sex-hormone claims research has appropriately emphasized the danger of drawing strong causal conclusions from imprecisely measured exposures [41].

Bone density and fracture risk

The effects of testosterone on bone provide an important contrast to the tendon literature. Testosterone treatment increases bone mineral density in hypogonadal men, with the largest effects generally seen in trabecular bone and the lumbar spine. In the Bone Trial of the Testosterone Trials, one year of testosterone gel increased spine volumetric bone density and estimated bone strength compared with placebo [42]. These changes are biologically and clinically meaningful, particularly in men with established androgen deficiency.

Improved density has not translated into proven fracture prevention. In the fracture substudy of the TRAVERSE trial, 3.50% of men assigned to testosterone experienced a clinical fracture compared with 2.46% assigned to placebo, corresponding to a hazard ratio of 1.43 [43]. Many fractures were associated with trauma or falls rather than classic spontaneous osteoporotic mechanisms. The finding was unexpected and does not necessarily indicate that testosterone weakens bone. It is also noteworthy that hypogonadism can be a cause of osteoporosis.

Several explanations remain possible, including chance, changes in activity, falls, risk-taking, or neuromuscular factors not captured by bone density. A meta-analysis of randomized trials found improvements in several musculoskeletal measures but did not establish a reduction in fractures [44]. Bone density, tendon integrity, muscle strength, balance, and exposure to trauma should therefore be considered related but distinct outcomes.

Clinical implications

The existing evidence does not justify withholding appropriately indicated testosterone therapy solely because of concern about tendon rupture. Major tendon injuries remain uncommon, and the available studies cannot determine how much of the observed association is pharmacologic. The most defensible interpretation is that a small increase in risk is plausible, with potentially greater relevance among highly active patients, those pursuing rapid strength gains, and those exposed to supraphysiologic concentrations.

Counselling should be proportionate. Patients do not need to be told that testosterone commonly causes tendon rupture. They can be informed that several observational studies have reported a small association, while the cause and magnitude remain uncertain. The distinction between replacement therapy and anabolic-androgenic steroid use should be made clear.

A baseline history of previous tendon rupture, chronic tendinopathy, recent fluoroquinolone exposure, corticosteroid use, smoking, diabetes, and planned training changes may help identify patients who warrant more detailed discussion. There is no evidence supporting routine tendon imaging before treatment.

For previously inactive patients, gradual progression of resistance training is reasonable. Large simultaneous increases in load, volume, frequency, and exercise novelty should be avoided regardless of testosterone use. New focal tendon pain, swelling, weakness, bruising, or a sudden change in function should prompt assessment rather than continued loading. These recommendations reflect standard sports medicine principles and should not be presented as a testosterone-specific rehabilitation protocol.

There is currently no basis for choosing a topical formulation over an injectable formulation solely to protect tendons. Similarly, more frequent lower-dose injections cannot yet be claimed to reduce tendon risk. Avoiding sustained supraphysiologic concentrations is consistent with good replacement practice, but a tendon-specific target concentration has not been established.

Research priorities

Prospective research should begin with better exposure measurement. Studies need verified diagnostic criteria, prescribed dose, formulation, injection frequency, adherence, and serial testosterone concentrations obtained at interpretable points in the dosing cycle. Exposure above the reference range should be analyzed separately from physiologic replacement.

Physical activity must be measured rather than assumed. Training age, weekly volume, intensity, major exercise changes, occupational loading, and participation in strength or contact sports are likely to be important effect modifiers. Baseline tendon symptoms and previous injuries should also be recorded.

Outcomes should distinguish tendinopathy, partial tearing, complete rupture, and surgical repair. Imaging-confirmed events and adjudicated injury mechanisms would improve specificity. Prospective ultrasound or magnetic resonance studies could examine tendon cross-sectional area, stiffness, strain, vascularity, collagen turnover markers, and regional differences before and after treatment.

A useful design would compare men with confirmed hypogonadism beginning treatment with untreated hypogonadal controls and eugonadal controls, while recording training exposure. Randomization by testosterone formulation or injection frequency could examine pharmacokinetic hypotheses, although tendon rupture would be too uncommon to serve as the sole primary outcome. Intermediate tendon measures would be more feasible.

Finally, supraphysiologic exposure should be studied as a separate category. Combining medically supervised replacement with nonmedical anabolic-androgenic steroid use obscures the likely importance of dose and training behavior. The question is not simply whether “testosterone” affects tendons, but under which biological and mechanical conditions a clinically meaningful risk emerges.

## Conclusions

Prescription testosterone has been associated with tendon tears and repairs across several anatomical sites. The consistency of the signal makes complete dismissal of the risk inappropriate, but the current evidence does not establish causation. Absolute event rates are generally low, the largest relative estimates arise from rare or imperfectly defined outcomes, and the major studies lack information about dose, testosterone concentration, physical activity, prior tendon disease, and nonprescribed androgen exposure.

Evidence from anabolic-androgenic steroid users and animal experiments provides biological plausibility and evidence of tendon risk. High androgen exposure may alter tendon stiffness, elongation, collagen organization, and matrix remodelling, while muscle strength and loading capacity can increase rapidly. These findings are not directly transferable to physiologic replacement, and a human dose-response relationship has not been fully demonstrated.

No evidence currently shows that topical therapy, smaller injections, or more frequent administration lowers tendon injury risk. Formulation-specific claims should therefore be avoided. For most appropriately selected patients, any causal increase in absolute risk is likely to be small. Clinicians should maintain physiologic treatment goals, discourage supraphysiologic exposure, and emphasize gradual loading progression in active patients without creating disproportionate concern about a rare and incompletely understood adverse outcome.
